# Emotion reactivity-related brain network analysis in generalized anxiety disorder: a task fMRI study

**DOI:** 10.1186/s12888-020-02831-6

**Published:** 2020-09-02

**Authors:** Jian Li, Yuan Zhong, Zijuan Ma, Yun Wu, Manlong Pang, Chiyue Wang, Na Liu, Chun Wang, Ning Zhang

**Affiliations:** 1 Nanjing Brain Hospital Affiliated to Nanjing Medical University, No 264, Guangzhou Road, Nanjing, 210029 China; 2 School of Psychology, Nanjing Normal University, Nanjing, 210097 China; 3 Department of Psychology and Behavioral Sciences, Zhejiang University, Hangzhou, 310007 China

**Keywords:** Generalized anxiety disorder, Emotion reactivity, Brain network analysis, Top-down control, Task-based fMRI

## Abstract

**Background:**

Generalized anxiety disorder (GAD) is closely associated with emotional dysregulation. Patients with GAD tend to overreact to emotional stimuli and are impaired in emotional regulation. Using emotional regulation task, studies have found hypo-activation in prefrontal cortex (PFC) of GAD patients and concluded with inadequate top-down control. However, results remain inconsistent concerning PFC and limbic area’s reactivity to emotional stimuli. What’s more, only a few studies aim to identify how limbic area interacts with PFC in GAD patients. The current study aims to identify the difference in PFC-limbic circuitry response to emotional stimuli between GAD patients and healthy controls (HCs) from the perspective of brain network. Through brain network analysis, it revealed the connectivity between limbic area and PFC, and moreover, the orientation of connectivity, all of which gave a better test of inadequate top-down control hypothesis.

**Methods:**

During fMRI scanning, participants were required to complete an emotional face identification task (fearful, neutral, happy facial expression). 30 participants (16 GAD patients, 14 HCs) were included in the formal analysis. A Bayesian-network based method was used to identify the brain network consisting of several pre-hypothesized regions of interest (ROIs) under each condition (negative, positive, neutral). In total, six graphs were obtained. Each of them represented the brain network that was common to the group under corresponding condition.

**Results:**

Results revealed that GAD patients showed more bottom-up connection but less top-down connection regardless of condition, relative to HCs. Also, the insula was more connected but the amygdala was less connected regardless of condition, relative to HCs. the results also revealed a very different brain network response between GAD patients and HCs even under neutral condition.

**Conclusions:**

More bottom-up connection but less top-down connection may indicate that GAD patients are insufficient in top-down control, in keeping with inadequate top-down control hypothesis. The more connected insula may indicate GAD patients’ abnormality in interoception processing. Relative to HCs, distinct brain network response pattern in GAD patients under neutral condition suggests GAD patients’ abnormality in distinguishing safety from threat and intolerance of uncertainty.

## Background

Generalized anxiety disorder (GAD) is thought to be one of the least successfully treated psychiatric disorders, which is largely attributable to its unclear neurobiological basis. Patients with GAD demonstrate pervasive, sustained, uncontrollable worry as their major symptomatic complaint [1, 2] and this is closely associated with emotional dysregulation [3, 4]. Neuroimaging and clinical studies in patients with GAD have contributed to the emotion dysregulation model [3]. The emotion dysregulation model holds that patients with GAD are inclined to overreact to both negative and positive emotional stimuli and exhibit abnormalities in emotion regulation [3, 4].

fMRI studies using emotional task have consistently found that abnormalities in prefrontal cortex (PFC)-limbic area in patients with GAD implicate in GAD’s pathology [5, 6]. According to emotional dysregulation model, patients with GAD tend to overrespond to emotional stimuli, but particularly, negative stimuli (e.g. heightened emotional intensity) [3, 4]. Relevant studies have mainly been conducted by using emotion processing task. Many studies have indicated that compared with healthy controls (HCs), GAD patients tended to over-activate PFC and/or cingulate cortex while confronting angry faces [7, 8], rating fearful faces [9], processing emotional images [10] and fearful pictures [11]. Though most of the extant studies using emotional processing task are consistent with this general picture, there are also some inconsistencies. For example, study has found that GAD patients were attenuated in PFC and anterior cingulate cortex (ACC) response to emotional stimuli [12]. Hyper-activation has also been reported in amygdala while GAD patients faced angry faces [7] and rated fearful faces [9], but with inconsistency too. For instance, studies have found activation in amygdala reduced [8] or no difference [12] while processing fearful faces in GAD patients, compared with HCs. Besides, there are inconclusive results too when it comes to the functional connectivity between amygdala and PFC, which was either decreased [7], increased [13] or no difference [9] in GAD patients, relative to HCs under emotional processing task. Similarly, mixed results have also displayed in functional connectivity between amygdala and cingulate area [10, 11]. In detail, one study has found that functional connectivity between posterior cingulate cortex (PCC) and amygdala was decreased [10], while another has found increased [11].

Emotional dysregulation model also predicts that GAD patients are incapable of appropriately regulating their emotions (e.g. maladaptive emotional management) [3, 4]. This has been mostly tested by emotion-regulation related task. Using reappraisal task (in which participants were required to maintain, downregulate or upregulate emotional response to emotional pictures), one study has found that GAD patients displayed reduced activation in PFC (e.g. dorsolateral PFC and dorsomedial PFC) during both reappraisal (reduce emotional response) and maintenance condition [14]. Consistent with this, another study has also found GAD patients displayed reduced activation in PFC (e.g. dACC) in Downregulate trials relative to the View trials [15]. Both studies have concluded that PFC’s hypo-activation found in GAD patients during emotional regulation task demonstrated their insufficient top-down control of limbic area [14, 15]. In line with this conclusion, another two studies have found similar results in GAD patients while required to make emotional adaptation. Etkin et al. [16] has found that GAD patients failed to activate ACC and a negative top-down ACC-amygdala connectivity under emotional regulation task, and this results was replicated in another study using the same paradigm by Etkin and Schatzberg [17], in which they have also found reduced ACC activation and decreased connectivity between ACC and amygdala.

Emotional dysregulation includes two separated, but mutually interplayed, abnormalities: abnormal emotional reactivity and regulation of reactivity [18]. Studies (as indicated above) have demonstrated that GAD patients displayed inadequate top-down control while emotion-regulation is required. This is also supported by review studies [5, 19] and meta-analytic study [20]. As to the origin of inadequate top-down control (in other words, PFC’s hypo-activation), it has been suggested that over-responsiveness of limbic circuitry could be the contributor that leads to the fatigue of top-down system (e.g. PFC), which makes the PFC unable to exert top-down control when needed [5, 14]. However, studies using emotional processing task appears to be in favor of overactive top-down control hypothesis, as indicated. What’s more, extant studies have fallen short of providing more direct evidence to this hypothesis. For one thing, results lack of consistency when it comes to PFC and limbic area’s reactivity to emotional stimuli (e.g. amygdala and its connectivity with other regions, as indicated above) [5]; secondly, very few studies aimed to identify how limbic area interacts with PFC. This makes it hard to interpret whether it’s inadequate/overactive top-down control in GAD. Therefore, for better understanding GAD’s pathology, more effort should be dedicated to clarifying GAD patients’ abnormal reactivity in PFC-limbic circuitry to emotional stimuli.

### The current study

This study aimed to identify the abnormal reactivity in the PFC-limbic circuitry of GAD patients under emotional face (happy, neutral, fearful) identification task from the perspective of brain network response. Through brain network analysis, the connection and the orientation of connection between brain regions were determined, which put the relations between PFC and limbic area into straight testing, adding evidence to inadequate/overactive top-down control hypothesis. Noteworthy, identifying the abnormalities in brain network response of GAD patients is important, for 1) it can deepen our understanding of GAD’s pathology: very few studies performed brain network analysis in GAD patients under emotional task, even for those did, most of them used functional connectivity analysis (which is correlation-based) to define brain networks, which normally took two brain regions at a time. When it comes to reveal how brain regions interacts with each other, correlation-based functional connectivity analysis is still facing some major challenges and having a long way to go (for detail, please see reference [21]). However, by brain network analysis, it not only can unveil how multiple brain regions interact with each other at the same time, but also can make causal inference between brain regions. Which can’t be done by general linear model (GLM) (GLM only addresses the level of response by multiple brain regions [22]); 2) it can be a powerful tool to differentiate different anxiety disorders: several studies have failed using GLM to identify GAD-specific neurobiological basis in compared with other anxiety disorders (e.g. panic disorder and/or social anxiety disorder) [23, 24]. However, as indicated above, brain network analysis can unveil how brain regions interact with each other, it can unearth potential differential response pattern in brain network between two groups even when GLM results are not significant .

Brain network analysis on fMRI data has, in recent years, gained significant interest, but current methods used on fMRI data have not performed well [25]. Smith et al. [25] has tested 38 extant methods used to identify networks on fMRI data and found none of them reached statistical significance with valid outcomes. A new algorithm called Multiple sample Greedy Equivalence Search (IMaGES) proposed by Ramsey et al. [26] has shown promise in identifying brain networks (finding and recalling connections) with excellent accuracy (over 95% in finding and recalling connections) [27]. When IMaGES is combined with an orientation algorithm named Linear non-gaussian Orientation Fixed Structure (LOFS), which can determine the direction of connections, it reaches a precision of over 90% in determining the direction of connections [27]. The combination of IMaGES and LOFS have been widely used in brain network analysis (please see reference [22, 28–32]).

One advantage of using IMaGES and LOFS is that it can include a dozen of brain regions when modeling their interactions without losing its performance. Besides, it needs no priori model to be set up like SEM or DCM [27]. Therefore, with the main focus on PFC-limbic circuitry, we specifically selected PFC, ACC, amygdala, insula and hippocampus as regions of interest (ROIs), upon which we performed brain network analysis. PFC, ACC and amygdala were all reported to act abnormally in GAD, as indicated above. Insula and hippocampus were also included because both insula and hippocampus have been found to be implicated in GAD’s pathology as well^1^ [1, 33, 34]. What’s more, all of these brain regions are considered as part of emotion circuitry [35].

We focused on comparing the orientation and density of connections in brain network between GAD patients and HCs. As there are no canonical brain network response pattern that can be used as a reference, we compared brain network characteristics of patients with those of HCs under each condition. Based on emotional dysregulation model and previous findings, we predicted: 1) more bottom-up (connections that originate from limbic area to prefrontal area) but less top-down connections in GAD patients versus HCs; 2) the connection strength between PFC and amygdala would be differential in two groups.

## Methods

### Participants

Patients with GAD and HCs were enrolled in this study. Groups were well matched on age and gender. All participants with GAD were consecutively recruited at the Department of Medical Psychology and Department of Mood Disorders of Nanjing Brain Hospital, affiliated with Nanjing Medical University. They were required to meet the following inclusion criteria: (1) A primary diagnosis of GAD by an experienced psychiatrist based on the Diagnostic and Statistical Manual of Mental Disorders (fifth edition, DSM-5TM); (2) Confirmation of GAD diagnosis using the Mini-International Neuropsychiatric Interview (MINI); (3) Free of psychiatric medications for at least 6 months prior to study enrollment; (4) Age 20–60 years old; (5) Right-handed and able to complete all study activities. Exclusion criteria for GAD participants were: (1) Having any neurological disorders affecting the central and/or peripheral nervous systems; (2) Any comorbid psychiatric disorders including depression, panic disorder, bipolar disorder, obsessive-compulsive disorder, schizophrenia, alcohol abuse and/or dependence, social phobia or eating disorder; (3) Severe physical illness, pregnancy and/or breastfeeding; (4) Suicide attempts in the past year; (5) Inability to complete MRI, and (6) A major life change in the last year as defined by death of spouse, unemployment, severe illness, serious injury, legal disputes, property loss, traffic accident, natural disasters or divorce.

HCs were recruited through online advertising and offline posters. Inclusion criteria included: (1) Age 20–60 years old; (2) Right-handed and able to complete all study activities. Exclusion criteria were: (1) Comorbid neurological disorders; (2) History of any symptoms consistent with a psychiatric disorder; (3) Pregnancy and/or breastfeeding; (4) History of psychological consulting or psychotropic medication within 3 months prior to study enrollment; (5) Inability to complete MRI; (6) Major life change in the last year as defined by death of spouse, unemployment, severe illness, serious injury, legal disputes, property loss, accidents, natural disasters or divorce.

### Task

Participants were required to identify emotional faces by pressing specific buttons corresponding to certain kinds of emotional faces (button “1” for negative, button “2” for neutral, button “3” for positive) while undergoing fMRI scanning. Emotional faces were all derived from the Chinese Affective Picture System (CAPS) [36]. Specifically, 20 images (10 were female, 10 were male) for each kind of emotion (happy, fearful, neutral) were randomly selected from its corresponding category (e.g. happy for positive, fearful for negative) for a total of 60 images. Of note, the keypad was used to ensure participants’ focus on the task. E-prime was used to program and present the emotional stimuli. A block design was employed for this study with each block consisting of 5 of the same kind of images. Each image was presented for 4 s. Before each block was presented, a 20-s cross was displayed on the screen. The entire task consisted of 12 blocks and lasted for 8 min in total. Images were not repeated. Balanced incomplete Latin square design and full Latin square design were used for within-participants and between-participants, respectively. Before the formal experiment, participants were well informed of the rules and practiced 3 times (images used in practice were not those that used in formal experiment).

### fMRI data acquisition

fMRI data was obtained on a 3.0 Tesla Siemens Medical System scanner at Nanjing Brain Hospital. In order to reduce head movement and noise, each participant’s head was secured with foam pads and earplugs were placed in ear canals. During the 8-min (240 volume, 36 slices for each volume) fMRI scan, each participant was instructed to perform the task they had practiced before scanning. Imaging data were acquired using the echo planar imaging (EPI) sequence according to the following scan parameters: Acquisition matrix = 64 × 64, field of view (FOV) = 240 mm × 240 mm, slice thickness = 4 mm, spacing between slices = 4 mm, 36 slices, repetition time (TR) = 2000 ms, echo time (TE) = 30 ms, flip angle (FA) = 90°.

### Data analysis

#### Imaging data processing

Imaging data were preprocessed using Data Processing Assistant Resting-State fMRI (DPARSF) software (http://rfmri.org/DPARSF) implemented in MATLAB. The basic procedures performed on images were slice timing, realignment, normalization to Montreal Neurological Institute (MNI) space with 3 mm × 3 mm × 3 mm resolution and smoothing with a 4 mm full width at half maximum (FWHM) Gaussian kernel. Participants were excluded if head motion exceeded 2.5 mm or 2.5 degrees. Individual and group level analysis were then performed using SPM8 software (http://www.fil.ion.ucl.ac.uk/spm).

#### Selection of ROIs

ROIs for brain network analysis were pre-hypothesized. We specifically focused on PFC-limbic circuitry, as we indicated previously. ROIs included PFC, ACC, amygdala, insula and hippocampus. All of them were found to implicate in emotional processing (e.g. fear) [1] and show abnormalities in GAD patients. ROIs were defined based on ALL (Automated Anatomical Labeling) atlas [37–39]. To identify clusters of ROIs that were significantly activated under different condition (positive, neutral, negative), one-sample t test was performed on all participants (GAD patients and HCs) under each condition to obtain condition-specific activation map. The significant activated clusters in pre-hypothesized ROIs were used as masks to extract timeseries for brain network analysis.

#### Brain network analysis

Brain network analysis was achieved with graphical causal modeling using IMaGES and the LOFS algorithm implemented in the TETRAD IV (version 5.3.0–1; http://www.phil.cmu.edu/projects/tetrad) software [27, 40]. Brain network analysis was conducted separately for each group under each condition (HC group under positive, neutral, negative condition; GAD group under positive, neutral, negative condition). Specifically, the time series of each ROI were extracted from each participant under each condition using REST [41] and were fed to IMaGES in order to identify the brain network shared by multiple participants under each condition. Unlike structural equation modeling (SEM) and dynamic causal modeling (DCM), IMaGES searched over all possible graphs (representatives of brain networks, in which nodes represent brain regions and connections between nodes represent connectivity between them) and through Bayesian Information Criterion (BIC) scoring, IMaGES picked the winning model with no need to posit a parametric model a priori [40]. IMaGES started with an empty graph for a set of nodes and took two stages to find the graph that was shared by multiple participants. Instead of searching over each and every directed acyclic graph (DAG), IMaGES searched over Markov equivalence classes to guarantee efficiency [26]. Since the data for brain network analysis was based upon an indirect measurement of neural activity and easily produced false triangles, IMaGES avoided spurious connections by increasing penalty discount [40]. Penalty discount was used to obtain the first graph with no triangulations [26]. At the forward searching stage, IMaGES added an additional connection (between nodes) at a time whose addition would most improve the BIC score. With each addition of a connection, the graph with the best BIC score would always be chosen [40]. When the addition of a connection no longer improved the BIC score, it initiated a backward searching stage where it eliminated, one at a time, connection whose removal most improved the BIC score. Once no further improvements can be made, it stopped and output a pattern (representative of the brain network) [26]. After IMaGES, the pattern was then submitted to LOFS to determine the connections’ direction for the graph by relying on the assumption that the residuals of the correct model with independent non-gaussian errors was doomed to be less Gaussian than the residuals of any incorrect model [25, 40]. The graph output by LOFS detailed the connections and the direction of the connections in the network which were described with arrows. In total, six graphs representing brain networks were obtained. In our study the Anderson–Darling score [42] was used to estimate the degree of non-Gaussianity while implementing LOFS. Afterwards, a structural equation modelling (SEM) estimator was employed to estimate the connection strength (represented by SEM coefficients) with a regression optimizer.

## Results

### Demographic analysis

In total, 42 participants (23 GAD patients, 19 HCs) were recruited for this study. Of them, two participants (one in the GAD group, one in the HC group) were excluded before data analysis because of poor data quality. For the remaining 40 participants, six GAD patients and four HCs were excluded for not meeting head motion criteria (less than 2.5 mm or 2.5 degrees). For the excluded participants, no significant difference on any characteristic (age: t = 1.5, *p* = 0.2; gender: χ^2^ = 0.17, *p* = 0.68). The remaining 30 participants were well matched on age and gender. See Table 1.

**Table 1:** Demographic and clinical characteristics of participants

	GAD	HC	Group difference
Gender, male/female	10/6	4/10	χ^2^ = 3.45, *p* = 0.06
Age (years) mean (SD)	35.25 (10.36)	40.05 (10.44)	t = 1.38, *p* = 0.18
HAMA, mean (SD)	19.56 (3.59)	N/A	
HAMD, mean (SD)	9.81 (3.58)	N/A	

*GAD* Generalized anxiety disorder, *HC* Healthy control, *HAMA* Hamilton anxiety rating scale, *HAMD* Hamilton depression rating scale, *SD* Standard deviation, *N/A* Not available, χ^2^ Pearson chi square

### fMRI between group analysis

Between group analysis found that abnormal activations in PFC, ACC, insula, hippocampus in GAD patients, relative to HCs, but no differences were found in amygdala’s activation between two groups (please see supplemental material Table 1).

### ROIs’ selection

**Fig. 1:**
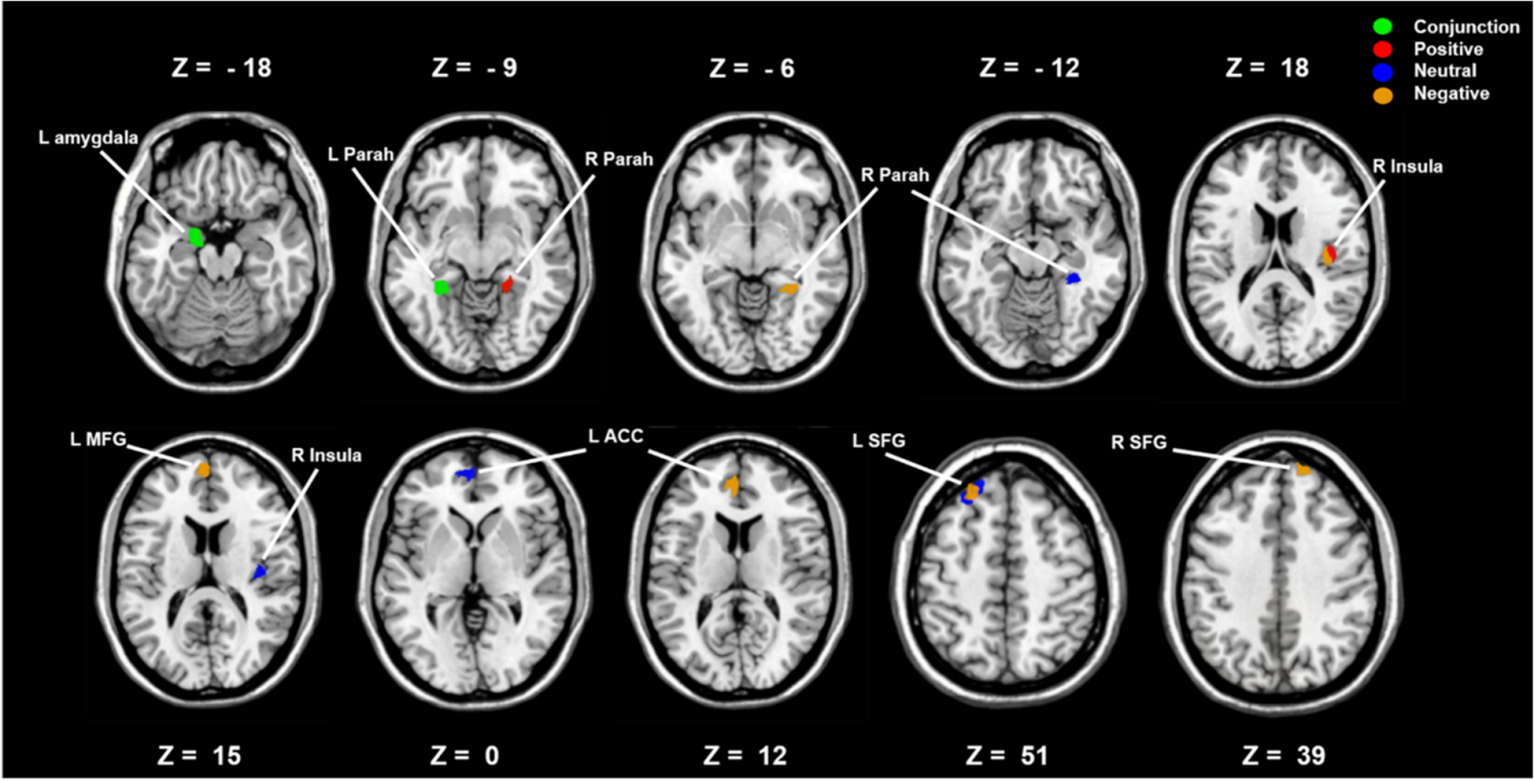
Significant activations in each pre-hypothesized regions of interest under different condition (corrected for multiple comparisons with voxel-wise FDR correction *p* < 0.05); different color designates activated clusters under different condition (red for positive, deep blue for neutral, yellow for negative, green for clusters activated across condition); LAmygdala = left amygdala, LACC = left anterior cingulate cortex, Rinsula = right insula, LParah = left parahippocampus, RParah = right parahippocampus, LMFG = left medial frontal, LSFG = left superior frontal gyrus, RSFG = right superior frontal gyrus

There were significant activations in each pre-hypothesized ROI (Table 2 and Fig. 1) (corrected with voxel-wise FDR correction *p* < 0.05). the peak coordinates for clusters were: right superior frontal gyrus (RSFG) (MNI: − 24, 42, 51; t = 5.05), left superior frontal gyrus (LSFG) (MNI: 15, 57, 39; t = 3.78), left anterior cingulate gyrus (LACC) (MNI: − 3, 48, 12; t = 4.59), right insula (RInsula) (MNI: 39, − 15, 18; t = 4.53), left medial frontal gyrus (LMFG) (MNI: − 3, 60, 15; t = 3.45), right parahippocampal gyrus (RParah) (MNI: 33, − 42, − 6; t = 5.99), left parahippocampal gyrus (LParah) (MNI: − 33, − 39, − 9; t = 5.99) and left amygdala (LAmygdala) (MNI: − 18, − 6, − 18; t = 3.91) under negative condition; LACC (MNI: − 3, 54, 0; t = 4.57), LSFG (MNI: − 24, 33, 51; t = 3.93), RInsula (MNI: 39, − 18, 15; t = 5.40), LParah (MNI: − 33, − 42, − 9; t = 5.04), RParah (MNI: 33, − 39, − 12; t = 5.43) and LAmygdala (MNI: − 21, 0, − 18; t = 3.90) under neutral condition; RInsula (MNI: 42, − 15, 18; t = 4.83), LParah (MNI: − 27, − 42, − 9; t = 5.76), RParah (MNI: 18, − 42, − 9; t = 5.15) and LAmygdala (MNI: − 18, − 6, − 18; t = 4.99) under positive condition (Table 2; Fig. 1).

**Table 2:** significant activations in each pre-hypothesized region of interest

Condition	L/R	Brain region	Abbreviates	Peak MNI coordinates	T-max	voxels
				x, y, z		
negative	L	Superior frontal gyrus	LSFG	−24, 42, 51	5.05	51
	R	Superior frontal gyrus	RSFG	15, 57, 39	3.78	9
	L	Anterior cingulate gyrus	LACC	−3, 48, 12	4.59	10
	R	Insula	RInsula	39, −15, 18	4.53	42
	L	Medial frontal gyrus	LMFG	−3, 60, 15	3.45	19
	R	Parahipocampaus	RParah	33, −42, −6	5.99	42
	L	Parahipocampaus	LParah	−33, −39, − 9	4.88	16
	L	Amygdala	LAmygdala	−18, −6, − 18	3.91	13
neutral	L	Anterior cingulate gyrus	LACC	−3, 54, 0	4.57	38
	L	Superior frontal gyrus	LSFG	−24, 33, 51	3.93	45
	R	Insula	RInsula	39, −18, 15	5.40	76
	L	Parahipocampaus	LParah	−33, −42, −9	5.04	27
	R	Parahipocampaus	RParah	36, −39, −12	5.43	28
	L	Amygdala	LAmygdala	−21, 0, −18	3.90	15
positive	R	Insula	RInsula	42, −15, 18	4.83	85
	L	Parahipocampus	LParah	−27, −42, −9	5.76	23
	R	Parahipocampus	RParah	18, −42, −9	5.15	34
	L	Amygdala	LAmygdala	−18, −6, −18	4.99	17

*MNI* Montreal Neurologic Institute, *L* Left, *R* Right; displaying results corrected for multiple comparisons with voxel-wise FDR correction *p* < 0.05

### Brain network analysis

**Fig. 2:**
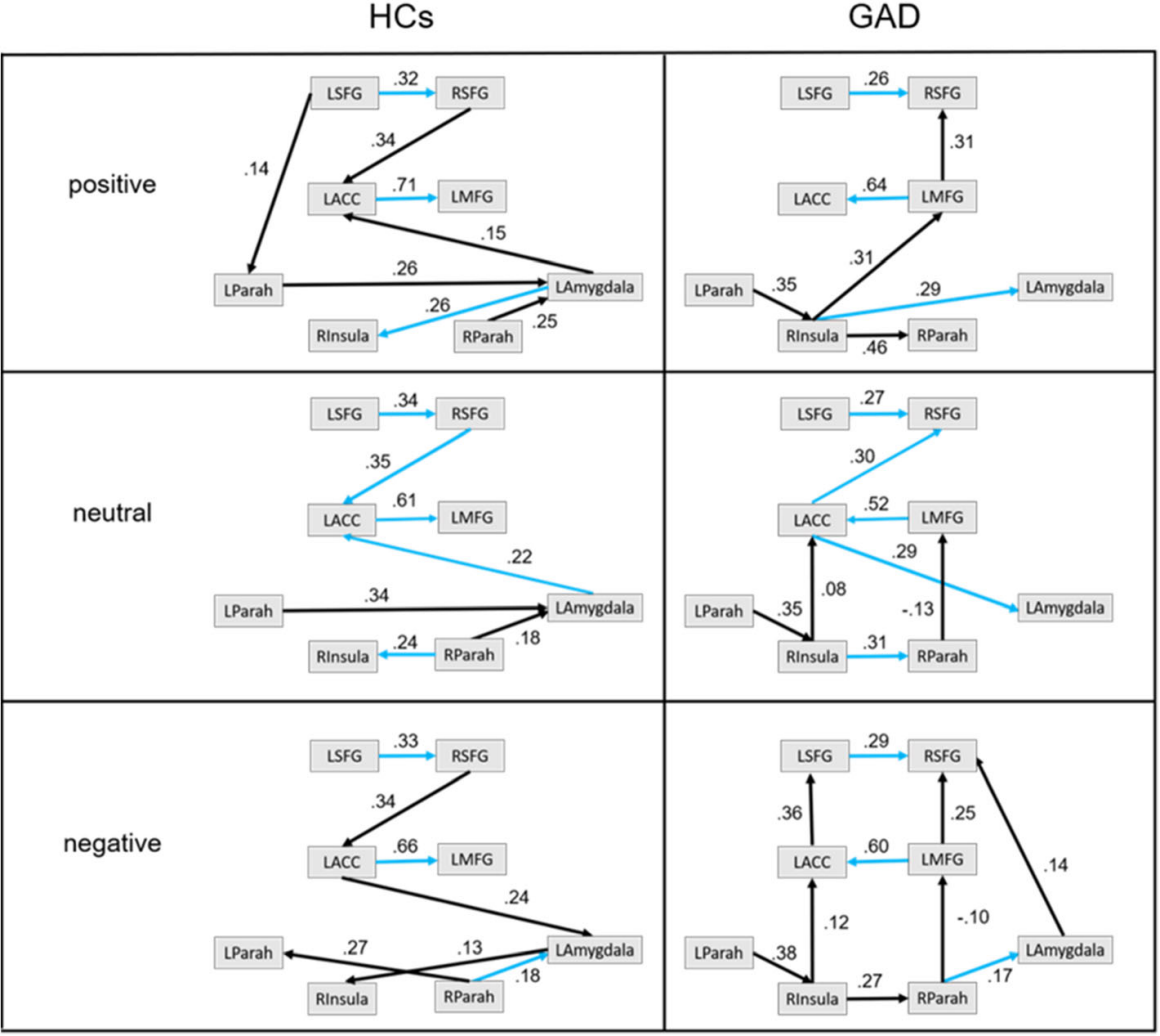
Brain network for two group under different condition; Arrow represents causal relation between ROIs; connections highlighted in blue were common to two groups under corresponding condition; numbers along with the connection were weight values; GAD = generalized anxiety disorder, HCs = health controls; LAmygdala = left amygdala, LACC = left anterior cingulate cortex, Rinsula = right insula, LParah = left parahippocampus, RParah = right parahippocampus, LMFG = left medial frontal, LSFG = left superior frontal gyrus, RSFG = right superior frontal gyrus

Six graphs were obtained in total. Each graph was chosen with the lowest BIC score (BIC = 112 for HCs under positive, BIC = 72 for HCs under neutral, BIC = 79 for HCs under negative and BIC = 115 for GAD under positive, BIC = 106 for GAD under neutral, BIC = 0 for GAD under negative). Each graph represented how brain regions interacted under corresponding condition. Orientation of the connections represented causal relations between ROIs. Numbers along with the connections represent the strength. The connections highlighted in blue were shared by two groups under corresponding condition. Connections common to two groups included: LSFG-RSFG, LACC-LMFG, RInsula-LAmygdala for positive condition; LSFG-RSFG, LACC-LMFG, RSFG-LACC, LACC-LAmygdala, RInsula-RParah for neutral condition; LSFG-RSFG, LACC-LMFG, RParah-LAmygdala for negative condition. None of them was significantly different in strength between two groups (two sample t test, all *p* values > 0.2). Connections common to all conditions in GAD group included: LSFG-RSFG, LMFG-LACC, LParah-RInsula, RInsula-RParah. Four separate Repeated measures ANOVA with condition as within-subjects variable showed a main effect of condition only for connection RInsula-RParah (F (2, 30) = 10.02, *p* < 0.0001, Partial Eta Squared = 0.40), but not for connection LSFG-RSFG (F (2,30) = 0.16, *p* > 0.5), connection LMFG-LACC (F (2,30) = 1.55, *p* > 0.1), connection LParah-RInsula (F (2,30) = 0.38, *p* > 0.5). for connection RInsula-RParah, subsequent comparisons revealed that under positive condition, it was stronger than that of under neutral condition and negative condition (positive vs neutral, t (15) = − 3.1, *p* < 0.01, d = 0.80; positive vs negative t (15) = − 4.48, *p* < 0.001, d = 1.15) (both passed a Bonferroni multiple comparisons correction). Connection common to both positive and negative condition in GAD group was LMFG-RSFG, no significant difference was found between two conditions (paired t test, t (15) = − 0.98, *p* > 0.05). Connections common to both neutral and negative condition in GAD group included: RInsula-LACC, RParah-LMFG. None of them was found significant different between two conditions (paired t test, RInsula-LACC, t (15) = 1.17, *p* > 0.1; RParah-LMFG, t (15) = 0.49, *p* > 0.1).

the number of connections is eight vs seven (HCs vs GAD) for positive condition, seven vs eight for neutral condition and seven vs ten for negative condition. The brain networks in GAD were denser than that of HCs under neutral and negative condition (densest under negative condition), but sparser than that of HCs under positive condition. There were always bottom-up connections in GAD group across condition (one of them under positive condition, two of them under neutral condition, three of them under negative condition), but only one top-down connection found under neutral condition. Under both positive and negative condition, top-down connections originating from LSFG to limbic area was found in HCs, while no top-down connection was found in GAD under any of two condition. Actually, the connection from LSFG was either disconnected with LParah under positive condition, or superseded by bottom-up connections when it reached to RSFG in GAD. There was one top-down connection in GAD under neutral condition (from ACC to LAmygdala), but this was not found in HCs. All the bottom-up connections in GAD were getting through the RInsula, then to PFC area across condition, which brought the Rinsula as a critical conjunction node in brain networks of GAD group. While in HCs, bottom-up/top-down connections were always getting through the LAmygdala. LACC was another critical conjunction node in brain networks of HCs across three conditions, with three or four connections connected (like the LAmygdala). While in GAD, it was either LMFG or LACC or both of them. LAmygdala in GAD was less connected across three condition (only one connection under neutral/positive condition, two under negative condition) relative to HCs. In PFC subnetwork, the orientation of connection between LACC and LMFG was reversed in GAD across conditions (Fig. 2).

### Exploratory analysis

**Fig. 3:**
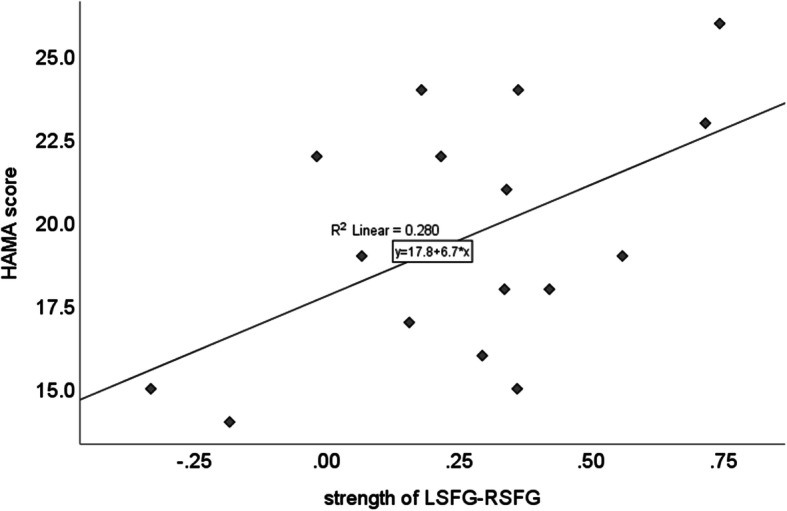
Correlation between HAMA score and the strength of LSFG-RSFG under positive condition. Pearson correlation coefficient = 0.53, *p* < 0.05.

Correlation analysis found that, under positive condition, the strength of LSFG-RSFG was positively correlated with HAMA score (Pearson correlation coefficient = 0.53, *p* < 0.05; see Fig. 3); No other correlation was found. (all *p* values were two tailed).

## Discussion

In this study, we used brain network analysis to identify the differential brain network response to emotional stimuli between GAD patients and HCs. The brain network comprised of several pre-hypothesized brain regions that were consistently reported having abnormal activation/functional connectivity in GAD patients during emotional tasks. Firstly, we replicated previous findings by GLM analysis that PFC area and limbic area were found abnormally activated in GAD patients during emotional tasks. Secondly, by brain network analysis, there were three main findings: 1) relative to HCs, GAD patients exhibited more bottom-up connections but less top-down connection; 2) regardless of condition, the insula was more connected, but the amygdala was less connected in GAD patients, relative to HCs; 3) GAD patients showed a different brain network response from HCs under neutral condition.

In keeping with our first prediction, we found more bottom-up but less top-down connection in GAD patients, relative to HCs. We concluded that this more bottom-up connection response pattern (less top-down but more bottom-up connection) in the brain network of GAD patients was likely to indicate inadequate top-down control. Specifically, under positive condition, what was common to GAD patients and HCs was that both groups had one bottom-up connection from limbic area to PFC. the bottom-up connections from limbic area to PFC during emotional task was highly likely to indicate an excitation process, since limbic area is the key part responsible for emotion generating (for example: the amygdala, insula and hippocampus [1, 43, 44]). However, besides the bottom-up connection, HCs also had one top-down connection from PFC (LSFG) to limbic area, which was not seen in GAD patients. The difference was even bigger under negative condition, in which GAD patients showed three bottom-up connections but no top-down connection, while HCs showed one top-down connection (LSFG to RSFG to LACC to LAmygdala). PFC is considered to be a key player in the cognitive modulation of emotion [35, 45] and its projecting to limbic area is considered critical to emotion modulation [46]. The top-down connections from LSFG to LAmygdala (to RInsula) shown in HCs under both positive and negative condition may demonstrate the cognitive modulation of emotion from PFC, preventing overresponse during emotional stimuli confronting [46]. Relatively, the lack of top-down connection and only having bottom-up connection in GAD patients under both negative and positive condition possibly indicated the imbalance between PFC’s cognitive modulation and limbic area’s emotional reactivity. More specifically, more bottom-up connection from limbic area but less top-down connection from PFC in GAD patients likely reflected the “over-responsiveness” of limbic area and PFC’s hypo-function, respectively. (note: over-responsiveness here meant denser connectivity from limbic area to PFC).

Across three conditions, the insula was more connected, but the amygdala was less connected in GAD patients, relative to that of HCs. The insula is associated with interoception generation from the body and is implicated in all kinds of subjective feelings (e.g. includes emotional awareness) [47]. Abnormal activation in insula is implicated in multiple kinds of anxiety disorders (e.g. social phobia [48], social anxiety disorder [24, 49]). Actually, it is proposed that inability to discriminate typical fluctuations in interoception from potential aversive body signal is key to anxiety disorder [50]. In GAD, it has reported abnormal connectivity between insula and amygdala under emotional task [9] or resting state [33]. GAD patients are characterized by excessive, uncontrollable and sustained worry, which is largely independent of environment [51]. Besides, they often have other clinical symptoms, like feeling on edge, muscle tension, etc. which are all integrated by insula to produce interoception and subject feelings. Therefore, it was reasonable to indicate that the enhanced-connectivity in insula was a reflection of the abnormal processing of interoception in GAD [50].

The amygdala was less connected regardless of condition in GAD patients, relative to HCs. The amygdala is implicated in emotional processing, emotional learning and cognitive evaluation of emotional stimuli and it is closely connected with cortical and subcortical regions [52, 53]. In HCs, the connections of amygdala with both subcortical and cortical regions was preserved. But in GAD patients, the amygdala was either disconnected with PFC (under positive condition), or disconnected with limbic area (under neutral condition), or less connected with limbic area (under negative condition), this may imply the dysfunction of amygdala in GAD patients like other studies [7, 13]), but from a perspective of brain network response. In detail, we utilized a more comprehensive and rigorous method to identify connections, a Bayesian network-based approach, in which determination of any connection between pairs of brain regions was assessed against the backdrop of the whole brain network [29]. This differed from the traditional correlation-based functional connectivity analyses as most studies used, which account for only two brain regions at a time and therefore can’t infer causal effect [25]. Therefore, correlation-based analysis may more easily find connections between two nodes, but they cannot be identified by using IMaGES.

Across condition, the insula directly/indirectly predicted the activity of amygdala in GAD patients, a reverse pattern relative to HCs. As discussed above, the over-connected of insula was implicated in GAD patients’ abnormal processing of interoception. The result that insula predicted amygdala’s activity may indicate that incapable of discriminately processing typical interoception and aversive body signal lead to increased emotional response [50].

Under neutral condition, the brain network response in GAD patients was different from that of HCs. Specifically, two bottom-up and one top-down connection were found in GAD patients. Patients with anxiety disorder is impaired in discriminating between threat and safety [54], tend to overgeneralize conditioned fear, respond to neutral stimuli as if the neutral stimuli are threat-related [55]. More bottom-up connections in GAD patients may indicate that GAD patients overgeneralized fearful reaction to nonthreatening stimuli, mistakenly treated the neutral human face as it was fearful, which triggered over-responsiveness in limbic area (reflected by the number of bottom-up connections). One top-down connection from ACC to amygdala was found too. ACC is implicated in emotional regulation and threat-related appraisal [16, 56]. However, we suggested the top-down connection under neutral condition was unlikely to reflect ACC’s regulation over amygdala’s activity. Specifically, in this subnetwork, it was the insula predicted ACC’s activity (directly/indirectly), then the ACC got to predict amygdala’s activity. Both connections from the insula to ACC may be caused by fear overgeneralization (as discussed above), therefore the ACC’s activity predicted by insula may implicate threat-related appraisal (rather than emotional regulation), which then progressively triggered amygdala’s activity. Consistent with this “not regulation” explanation, study using fear generalization paradigm has also found that GAD patients showed insufficient PFC regulation over fear-generalized stimuli [57].

Inconsistent with the second prediction, among the connections that were common to both GAD patients and HCs, we didn’t find any pair of connections differ in their strength. The connection between LSFG and RSFG was exactly the same between two groups (same connection with the same orientation), and it didn’t vary across condition. No significant difference in the strength of this connection between two groups may indicate the communication between LSFG and RSFG was preserved in GAD patients. However, this connection was positively correlated with HAMA score in GAD patients under positive condition. To the best of our knowledge, this was the first time that connectivity between LSFG and RSFG was reported to positively correlate with anxiety level. This added evidence to the significance of lateral PFC in clinical symptom. Regardless of condition, the LMFG was more connected in GAD patients, compared with LACC, a reverse pattern in HCs. The medial PFC is implicated in introspective thinking and a dysregulation of this region may underlie the uncontrollable worry in GAD patients [58].

Together with GLM results. In this study, the amygdala’s activity during tasks did not differ between GAD patients and HCs. No difference in amygdala’s activity between two groups has been observed in previous studies [12, 59], it may need specific fearful stimuli to elicit abnormal amygdala response in GAD patients [12]. We didn’t find increased activity in amygdala during presentation of fearful faces in either group, which is somewhat at odds with previous studies [12, 60, 61]. Between group analysis revealed that both limbic area and PFC were under-activated in GAD patients regardless of condition. Integrated with the brain network response pattern, it may suggest that GAD patients’ brain network response pattern is rigid, showing more bottom-up connections even when the limbic area is not as active as in HCs.

Several limitations are important to note. First, the accuracy and reaction time of the behavioral results were not recorded in this study, as other studies [59, 62]. Lack of behavioral results may make the result less convincing than it should be. Second, the relatively small sample size. Twenty-three GAD patients and 19 HCs were recruited. However, because of poor data quality and excessive head motion during scanning by couple of participants, the final sample size was 16 GAD patients and 14 HCs and this may be the reason no other significant correlation between clinical measurements and connectivity was found. Last, no mood rating was administered with participants after scanning, which may confine the explanation of the result.

Based on the current results, there are several implications for future research. First, compared with other anxiety disorders (e.g. social anxiety disorder), less studies have found aberrations in the insula of GAD patients. However, in this study, we found a highly distinct role of insula in GAD patients during emotional tasks. As indicated, the insula is implicated in interoception and has close link with autonomic system [63], future studies may benefit from combining fMRI technique with physiological measurements. Second, in this study, brain network analysis provided several new insights on GAD’s pathology (e.g. the aberrations in the insula). However, GAD and other anxiety disorders (e.g. social anxiety disorder and panic disorder) have been reported to share similar underlying neural mechanism [23, 24]. For example, one study has found that increased amygdala activation was found in patients with different diagnosis (GAD or panic disorder or social anxiety disorder) during fearful faces processing [23]. This may imply over-response in limbic area is common to all kinds of anxiety disorder. However, in the same study they have also found patients with panic disorder showed a unique hyperactivation in the posterior insula and a shared dysregulation in temporal regions by patients with panic disorder or social anxiety disorder, an implication of disorder specific neural mechanism. In another study, researchers haven’t found GAD specific, nor panic-disorder specific neural mechanism during emotional regulation task. Both groups showed a hypoactivation in PFC, implying insufficient top-down control [24]. These two studies may indicate that different anxiety disorders share the same hypoactivation in PFC and overresponse in limbic area. But in the meantime, they also have disorder specific neural mechanism (like the insula). Hence, it is important for future studies to use the same kind of brain network analysis to identify disorder-common and -specific circuitry among several anxiety disorders, for it not only can better identify different anxiety disorders based on neuroimaging techniques, but also can drive more specialized treatment to each disorder.

Third, by including neutral condition in the brain network analysis, we found evidence to fear overgeneralization hypothesis concerning GAD patients. However, in addition to fear overgeneralization, findings of more bottom-up connections in GAD patients under neutral condition also aligned well with the prediction of intolerance of uncertainty. Studies found GAD patients are intolerant of uncertainty [64], tend to have negative feelings while facing uncertainty [65] and reduction in the score of intolerance of uncertainty often predicted the improvements of GAD symptoms [66, 67]. Neutral facial expressions are the most ambiguous of all the stimuli and more likely to be perceived as negative even in health controls [68], study has found. Hence, undoubtedly, intolerance of uncertainty in GAD patients could also be a main contributor to the findings under neutral condition. Last, neutral facial expressions condition was often treated as “neutral” in previous studies of GAD. However, the current study provided direct evidence against that setting, at least from the perspective of neuroimaging.

## Conclusion

Through brain network analysis, we found that relative to HCs, GAD patients exhibited more bottom-up connections but less top-down connection in their emotional reactivity brain network, which may indicate inadequate top-down control. In addition, compared with HCs, the insula was more connected with other brain regions in GAD patients, which may indicate abnormal interoception processing in GAD patients. Lastly, brain network response was different between GAD patients and HCs under neutral condition, possibly an indicative of fear overgeneralization and intolerance of uncertainty in GAD patients.

## Supplementary information

**Additional file 1: Supplemental material Table 1**. between group activation during emotional task. **Supplemental material Table 2**. within group activation duration emotion task

## Data Availability

The task fMRI datasets used during the current study are available from the corresponding author on reasonable request.

## Abbreviations

GAD: Generalized anxiety disorder
HCs: Healthy controls
PFC: Prefrontal cortex
fMRI: Functional magnetic resonance imaging
ACC: Anterior cingulate cortex
IMaGES: Multiple sample Greedy Equivalence Search
LOFS: Linear non-gaussian Orientation Fixed Structure
ROI: Region of interest
HAMA: Hamilton anxiety rating scale
HAMD: Hamilton depression rating scale
L/RSFG: Left/right Superior frontal gyrus
LMFG: Left Medial frontal gyrus
L/RParah: Left/right Parahippocampus
LInsula: Left insula
LACC: Left ACC
LAmygdala: Left amygdala

## Footnotes

1 Firstly, hippocampus is considered part of the limbic system responsible for fear generation and was part of common neurocircuitry implicated in all anxiety disorders [1]; secondly, connectivity between insula and amygdala was impaired in GAD patients [9, 33]; thirdly, both insula and hippocampus showed increased activation under emotional task in GAD patients [34].
