# Supplementary material for: Emotion reactivity-related brain network analysis in generalized anxiety disorder: a task fMRI study
Source: BMC Psychiatry. 2020 Sep 2;20:429. doi: 10.1186/s12888-020-02831-6 (PMC7466835; doi:10.1186/s12888-020-02831-6)
Supplement: Supplementary file 1 — Additional file 1: Supplemental material Table 1. between group activation during emotional task. Supplemental material Table 2. within group activation duration emotion task [file 12888_2020_2831_MOESM1_ESM.docx]

Supplemental material table 1. between group activation during emotional task

| Condition | Comparison | Brain region | L/R | Peak MNI coordinates  (x, y, z) | voxels | T-max |
| --- | --- | --- | --- | --- | --- | --- |
| Positive (vs neutral) | GAD < HCs | Superior frontal gyrus | R | 24, 45, 36 | 133 | 3.11 |
|  |  | Inferior parietal lobe | R | 63, -42, 36 | 214 | 3.3 |
|  |  | Middle frontal gyrus | L | -45, 15, 36 | 110 | 2.69 |
|  |  | Hippocampus | L | -24, -21, -12 | 115 | 3.48 |
|  |  | Inferior parietal lobule | L | -57, -36, 48 | 162 | 3.09 |
|  |  | Middle temporal gyrus | R | 60, -36, -12 | 175 | 3.37 |
|  | GAD > HCs | Postcentral gyrus | R | 45, -21, 48 | 216 | 3.02 |
|  |  | Superior temporal gyrus | R | 69, -30, 12 | 216 | 3.86 |
|  |  | Caudate | R | 15, 21, -6 | 450 | 4.02 |
| Negative (vs neutral) | GAD < HCs | Superior frontal gyrus | R | 15, 12, 54 | 254 | 3.87 |
|  |  | Superior frontal gyrus | L | -18, 3, 69 | 168 | 4.05 |
|  |  | Anterior cingulate cortex | R | 6, 33, 15 | 161 | 3.6 |
|  |  | Anterior cingulate cortex | L | 0, 36, 3 | 153 | 2.64 |
|  |  | Insula | R | 33, -15, 21 | 131 | 3.9 |
|  |  | Insula | L | -39, 0, -9 | 129 | 3.42 |
|  |  | Middle cingulate gyrus | R | 12, -30, 39 | 161 | 2.85 |
|  |  | thalamus | R | 12, -30, 9 | 167 | 4 |
|  |  | Hippocampus | R | 36, -9, -15 | 138 | 3.8 |
|  |  | Middle cingulate gyrus | L | -12, -18, 42 | 153 | 3.83 |
|  |  | Postcentral gyrus | R | 18, -33, 81 | 332 | 5.29 |
|  |  | Postcentral gyrus | L | -18, -42, 69 | 173 | 4.31 |
|  |  | Precentral gyrus | R | 24, -18, 69 | 145 | 3.89 |
|  |  | Precentral gyrus | L | -21, -15, 69 | 178 | 3.76 |
|  |  | Superior parietal lobule | R | 24, -54, 63 | 112 | 2.91 |
|  |  | Inferior parietal lobule | R | 48, -33, 39 | 215 | 4.11 |
|  |  | Precuneus | L | -6, -51, 75 | 112 | 2.33 |
|  |  | Middle temporal gyrus | L | -45, -60, 6 | 109 | 3.07 |
|  | GAD > HCs | Inferior frontal gyrus | R | 54, 30, 3 | 149 | 3.19 |

Notes: HCs = healthy controls; GAD = generalized anxiety; MNI = Montreal Neurologic Institute; L = left; R = right. Displaying results corrected for multiple comparisons across the entire brain (individual voxel *p* < 0.05, AlphaSim corrected with *p* < 0.01, cluster size > 108).

Supplemental material table 2. within group activation duration emotion task

| Group | Condition (vs neutral) | Brain region | L/R | Peak MNI coordinates  (x, y, z) | voxels | T-max |
| --- | --- | --- | --- | --- | --- | --- |
| HCs | Positive | Medial frontal gyrus | R | 6, 42, 33 | 24 | 5.99 |
|  |  | Inferior frontal gyrus | R | 60, -42, -12 | 13 | 4.28 |
|  | Negative | Middle frontal gyrus | L | -42, 18, 33 | 58 | 6.40 |
|  |  | Postcentral gyrus | R | 24, -30, 57 | 320 | 4.16 |
|  |  | Anterior cingulate cortex | L | -3, 27, 15 | 55 | 4.57 |
|  |  | Medial frontal gyrus | L | -6, 18, 42 | 28 | 5.42 |
|  |  | Inferior frontal gyrus | L | -60, 15, 24 | 10 | 4.16 |
|  |  | insula | R | 33, 12, 6 | 63 | 4.14 |
|  |  | insula | L | -33, 12, 6 | 20 | 4.47 |
|  |  | Middle frontal gyrus | R | 48, 12, 45 | 51 | 3.84 |
|  |  | Superior frontal gyrus | R | 15, 33, 42 | 36 | 4.95 |
| GAD | Positive | Precentral | R | 36, -27, 57 | 12 | 3.76 |
|  |  | Superior temporal gyrus | R | 69, -30, 12 | 14 | 4.46 |
|  |  | Inferior parietal lobule^1^ | L | -60, -42, 36 | 27 | 3.80 |
|  |  | Thalamus^1^ | L | -9, -30, 3 | 24 | 3.83 |
|  | Negative | Middle temporal gyrus | R | 63, -39, -15 | 11 | 4.22 |
|  |  | Hippocampus^1^ | L | -33, -27, -12 | 12 | 4.77 |
|  |  | Postcentral^1^ | L | -24, 36, 72 | 15 | 3.57 |

Note: ^1^ vs neutral decreased. uncorrected p < 0.005 cluster size > 10. HCs = healthy controls; GAD = generalized anxiety; MNI = Montreal Neurologic Institute; L = left; R = right.
